# Epigenetic Landscape of Female Infertility: An Integrated Bioinformatics Perspective on DNA Methylation, MicroRNAs, and Gene Regulatory Networks Across PCOS, Endometriosis, and Diminished Ovarian Reserve

**DOI:** 10.3390/ijms27041785

**Published:** 2026-02-12

**Authors:** Maroua Jalouli, Md Ataur Rahman, Saber Nahdi, Abdel Halim Harrath

**Affiliations:** 1 Department of Biology, College of Science, Imam Mohammad Ibn Saud Islamic University (IMSIU), Riyadh 11623, Saudi Arabia; 2 Department of Oncology, Karmanos Cancer Institute, Wayne State University, Detroit, MI 48201, USA; 3 Department of Zoology, College of Science, King Saud University, Riyadh 11451, Saudi Arabia

**Keywords:** female infertility, DNA methylation, microRNA, epigenetics, bioinformatics, PCOS, endometriosis, ovarian reserve

## Abstract

Female infertility diseases such as polycystic ovary syndrome (PCOS), endometriosis, diminished ovarian reserve (DOR), and recurrent implantation failure (RIF) have different clinical phenotypes. However, they might be epigenetically convergent, and thus the therapeutic targets may be potential. This study utilized transcriptome data, microRNA (miRNA), and DNA methylation data from the granulosa cells of four Gene Expression Omnibus (GEO) datasets, GSE138518, GSE105765, GSE232306, and GSE92324, to conduct integrated bioinformatics analysis. We focused on differentially expressed genes (DEGs), constructed a miRNA–mRNA network, performed ROC curve analysis, and conducted function enrichment and drug repurposing studies. Our findings identified eight dysregulated genes (H19, SULT1A4, HCK, SPI1, CARD16, NFE2, LST1, and KRT8) common to PCOS, DOR, and RIF, which may serve to distinguish PCOS specifically. Moreover, these DEGs are associated with pathways such as innate immune activation, inflammatory responses, the NOD-like receptor signaling pathway, and Fc gamma R-mediated phagocytosis. Notably, MiRNAs differentially expressed in endometriosis (specifically hsa-miR-202-5p and hsa-miR-141-3p) were found to directly target this gene set, highlighting the role of epigenetic regulation across infertility diseases. Additionally, our drug repurposing analysis identified several FDA-approved drugs, including Abacavir and Peginterferon Alfa-2b, suggesting that the HCK gene may be a viable target for drug development to address female infertility. Furthermore, we identified 192 genes that correlated with DNA methylation and expression levels in PCOS. Thus, this study underscores the epigenetic convergence of different female infertility diseases and highlights potential biomarkers and therapeutic options that could enhance treatment in reproductive medicine.

## 1. Introduction

Infertility is a global problem, affecting up to 10–15% of couples. Female infertility accounts for over 50% of cases [1]. Despite a broad range of clinically distinct disorders such as polycystic ovary syndrome (PCOS), endometriosis, diminished ovarian reserve (DOR), and recurrent implantation failure (RIF), they often have shared pathophysiological mechanisms, which include chronic inflammation, hormonal dysregulation, and aberrant tissue remodeling [2,3,4]. While much of the study of these disorders has focused on genetic and anatomical predisposition in isolation, there remains a large proportion of unexplained cases, resulting in greater interest in dynamic and regulatory mechanisms such as epigenetics [5].

Epigenetic processes regulate gene expression and may be heritable but do not involve alterations to the DNA sequence. Epigenetic mechanisms that have been linked to reproductive function and/or infertility in females include DNA methylation, histone modifications, and microRNA expression [6]. DNA methylation is generally associated with transcriptional silencing, and abnormal methylation patterns can result in aberrant silencing of genes important for folliculogenesis, steroidogenesis, and endometrial receptivity [7]. MicroRNAs (miRNAs) are another epigenetic process that regulates gene expression by mediating mRNA degradation or translational repression. Altered miRNA expression has been associated with the development of all the major female infertility conditions [8,9].

Nonetheless, a significant caveat exists. The overwhelming majority of these studies (as well as in most research involving “omics” data) have focused on the “single-omics, single-disorder” model. This approach may blind researchers to cross-regulatory circuitries that may not be mutually exclusive regarding infertility diagnoses. Such a myopic view of the infertility “omics” universe may have clouded our ability to detect the presence of convergent molecular pathways that may not only reveal shared biological etiologies but also lead to common diagnostic and therapeutic endpoints. Integrative bioinformatics-based analyses that combine multiple-omics datasets (transcriptomic, miRNomic, and methylomic) may enable such system-level differences to be revealed, beyond clinical categories [10].

**Figure 1:**
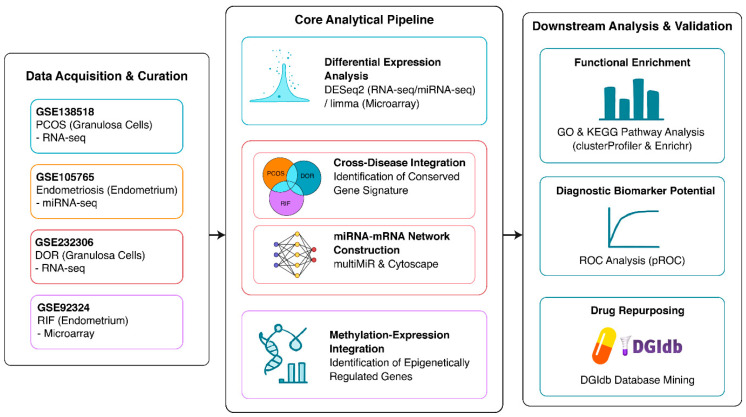
Study workflow from raw data acquisition through a multi-tiered analytical process to final analysis and validation.

This study addresses the research gap by conducting an integrative bioinformatics analysis of multiple infertility disorders. We retrieved data from four publicly available genome-wide datasets that profiled, in pathophysiologically relevant tissues (granulosa cells and endometrium), the epigenomic landscapes of four major causes of infertility: PCOS, endometriosis, DOR, and RIF. Moreover, the main goals of this study were to identify and confirm disorder-specific molecular signatures; to identify and functionally characterize a conserved gene network shared among the four diseases; and to assess the diagnostic and therapeutic significance of this global, integrated epigenetic landscape. To our knowledge, this study represents the first endeavor to transcend the established disease-centric framework to explore female infertility through the lens of common epigenetic disruptions. Figure 1 provides an overview of the study design and the key characteristics of the four datasets used, serving as a roadmap for the entire analysis.

## 2. Results

### 2.1. Dataset Overview and Quality Control

**Figure 2:**
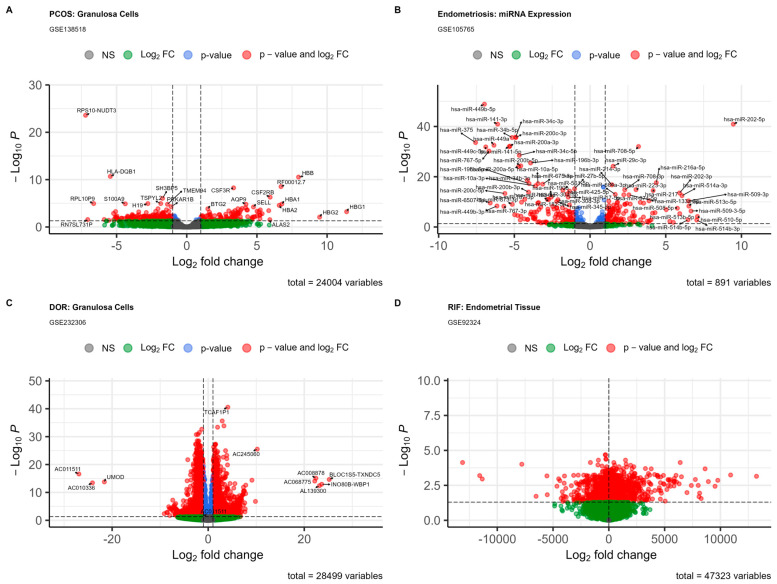
Gene Expression Profiles in Female Infertility Conditions. (**A**) In PCOS, granulosa cells display an aberrant expression of genes involved in metabolism and inflammation. (**B**) Endometriosis is characterized by specific miRNA expression profiles, such as that of miR-202-5p. (**C**) DOR is associated with changes in the expression of genes linked to ovarian reserve. (**D**) RIF is marked by dysregulation of genes related to implantation in endometrial tissue. Red points are significantly upregulated, and blue points are downregulated (*padj* < 0.05, |log2FC| > 1).

To provide a consistent framework for cross-disease integration, we subjected all four datasets to stringent quality control and preliminary exploratory analysis. PCA and hierarchical clustering consistently identified well-delineated case and control groups within each dataset, confirming the biological integrity of the cohorts and the technical quality of the omics data. Subsequent differential expression analysis generated detailed molecular signatures for each disorder (Figure 2). In PCOS granulosa cells (GSE138518), 304 DEGs (110 upregulated, 194 downregulated; |log_2_FC| > 1, FDR < 0.05) were identified, aligning with previous evidence of metabolic and inflammatory dysregulation in this tissue (Supplementary File S1). Endometriosis endometrium (GSE105765) exhibited 254 DEmiRs (131 upregulated, 123 downregulated). hsa-miR-202-5p, hsa-miR-708-5p, and hsa-miR-29c-3p were among the most significantly upregulated miRNAs, while hsa-miR-449b-5p, hsa-miR-141-3p, and hsa-miR-34c-3p were among the most downregulated (Supplementary File S2). DOR granulosa cells (GSE232306) displayed transcriptomic changes in genes associated with ovarian reserve and follicular development (Supplementary File S3). In RIF endometrium (GSE92324), we identified 2919 DEGs (1598 upregulated, 1321 downregulated), many of which are known to play essential roles in embryo implantation and endometrial receptivity (Supplementary File S4).

### 2.2. Cross-Disease Epigenetic Dysregulation Across Infertility Disorders

The lists of differentially expressed genes identified in all four infertility disorders were large, but not completely mutually exclusive (Figure 2). Within PCOS GSE138518, the most prominent features included extensive downregulation of genes linked to inflammation and cell identity (Figure 2A). Here, it was striking to see the concerted downregulation of several hemoglobin (*HBB, HBA1*, and *HBA2*) genes, which are now recognized to have roles other than oxygen transport, including responses to oxidative stress and metabolic homeostasis [11]. Thus, the possibility that such downregulation in PCOS may contribute to aberrant metabolic function or cellular stress in these cells cannot be ruled out. Interestingly, known inflammatory factors, including S100A9 and CSF3R, were also among the most upregulated genes in PCOS. This is consistent with the idea that PCOS is associated with chronic low-grade inflammation [12]. H19, a long non-coding RNA (lncRNA) associated with imprinting control and cell growth, was among the most dysregulated lncRNAs in PCOS [13].

The identified endometriosis miRNA profile (GSE105765) displayed a specific dysregulated epigenetic signature (Figure 2B). We explored a panel of significantly dysregulated miRNAs, including upregulated hsa-miR-202-5p, hsa-miR-708-5p, and hsa-miR-29c-3p and downregulated hsa-miR-449b-5p, hsa-miR-141-3p, and hsa-miR-34c-3p. These miRNAs are known to directly regulate cellular proliferation, inflammation, and remodeling processes. For example, the downregulation of miR-449 and miR-200 family members promotes epithelial–mesenchymal transition and fibrosis, processes implicated in endometriosis development [14,15].

Next, the analyzed DOR granulosa cells (GSE232306) showed a transcriptomic profile indicative of altered follicular function (Figure 2C). Dysregulated genes, such as SECISBP2L and UMOD, suggest perturbations in essential cellular pathways, including selenium metabolism and extracellular matrix composition, potentially influencing oocyte quality and follicular dynamics [16]. These alterations are characteristic of a molecular profile with diminished ovarian reserve.

Lastly, the identified RIF endometrial transcriptome (GSE92324) showed a significant dysregulation of genes known to be critical for endometrial receptivity (Figure 2D). The number of DEGs and the magnitude of fold-changes observed suggest a severely impaired window of implantation. Specific downregulation of cell adhesion, signaling, and immune-modulatory genes likely contributes to the defective embryo-endometrial communication observed in RIF.

### 2.3. Conserved Genes Immune Signature Reveals Common Pathophysiological Pathways

**Figure 3:**
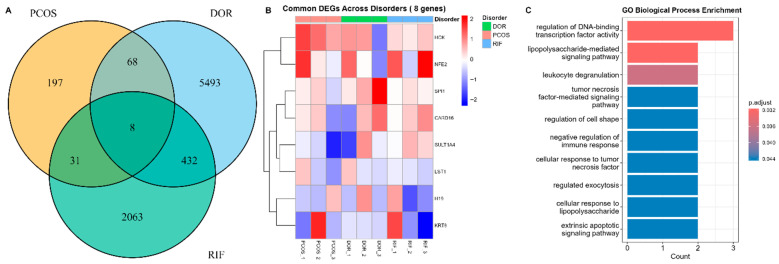
Epigenomic Convergence across Pathologies. (**A**) A Venn diagram illustrates the intersection of statistically significant DEGs among PCOS, DOR, and RIF, identifying conserved molecular mechanisms. (**B**) Heatmap depicting the expression patterns of the conserved DEGs across conditions, which shows a persistent pattern of dysregulation. (**C**) GO analysis of overlapping genes identifies enrichment of immune system regulation, vascular homeostasis, and inflammation.

The results of cross-disease integrated analyses are shown in Figure 3. To determine common pathways, Venn diagrams were constructed to identify sets of genes that were differentially expressed between PCOS and controls, DOR and controls, and RIF and controls (Figure 3A). A total of eight genes (H19, SULT1A4, HCK, SPI1, CARD16, NFE2, LST1, and KRT8) were identified as intersecting genes with altered expression across the three clinical disorders. A heatmap of these eight genes showed that they were similarly dysregulated across all three clinical disorders (Figure 3B).

Functional enrichment analysis of this eight-gene signature gave further deep insight into the shared pathophysiology. The Gene Ontology (GO) analysis for Biological Processes showed a highly significant overrepresentation of terms directly linked to immune dysregulation and inflammatory signaling (Figure 3C). The most significantly enriched terms were “cellular response to lipopolysaccharide”, “tumor necrosis factor-mediated signaling pathway”, and “negative regulation of immune response”, clearly implicating innate immune activation and cytokine-driven inflammation as the predominant mechanisms common to PCOS, DOR, and RIF [17,18,19]. Other terms such as “leukocyte degranulation” and “regulated exocytosis” indicate the activation of immune cells and the secretion of proteins.

The genes in this signature offer mechanistic plausibility for these enriched pathways. HCK and SPI1 are master regulators of myeloid cell function and inflammation [20,21]. CARD16 is an essential component of the inflammasome, a multiprotein complex that activates inflammatory cytokines [22]. LST1 is an adapter that influences immune cell adhesion and activation [23]. NFE2 is a transcription factor that controls oxidative stress response in hematopoietic cells [24]. The presence of KRT8, a cytoskeletal protein, and H19, a long non-coding RNA involved in cell growth and imprinting [25,26], implies that this shared pathology is not limited to basic immunology but is also at the nexus of more fundamental cellular integrity and developmental mechanisms.

### 2.4. Pathway Analysis Reveals a Convergent Theme of Immune Dysregulation and Altered Cellular Integrity

**Figure 4:**
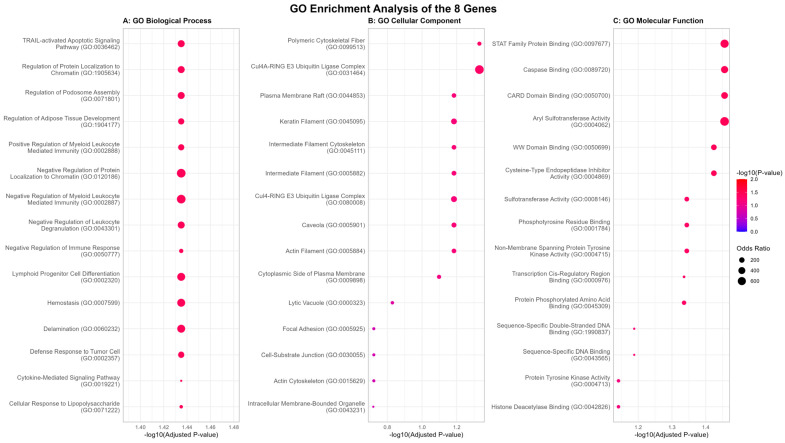
Gene Ontology (GO) Enrichment Analysis of Conserved Infertility Gene Signature. GO enrichment of eight genes overlapping PCOS, DOR and RIF in three GO categories. (**A**) Biological Process, showing enrichment in immune regulation, myeloid cell function, and cytokine signaling. (**B**) Cellular Component, showing association with cytoskeletal structures (keratin filaments, actin cytoskeleton) and membrane signaling complexes. (**C**) Molecular Function, showing enrichment in kinase activity, DNA binding, and protein–protein interaction domains. Dot size is proportional to the odds ratio, and color is proportional to the statistical significance (−log10 adjusted *p*-value).

To better understand the potential biological relevance of the eight-gene conserved signature genes, we next conducted a functional enrichment analysis. The results showed that the functions of this eight-gene conserved signature were significantly enriched in biological processes and molecular functions, primarily focused on immune inflammation and the cytoskeleton, consistent with the pathophysiological mechanisms shared by PCOS, DOR, and RIF. Gene Ontology (GO) enrichment of the three major categories of the eight-gene conserved signature showed a multi-dimensional role (Figure 4). First, from a biological processes perspective, most of the top significant enrichment terms were related to immune responses (Figure 4A). The top three terms were “negative regulation of immune response”, “positive regulation of myeloid leukocyte mediated immunity”, and “cytokine-mediated signaling pathway”, which further indicated the common pathophysiological mechanism of PCOS, DOR, and RIF disorders: excessive activation of innate immunity and inflammation [27]. In addition, processes such as “cellular response to lipopolysaccharide” and “defense response to tumor cell” were also significantly enriched, further suggesting a hyperactive immune surveillance state [28]. In addition, the most significant term “hemostasis” indicates a certain association with abnormal vascular and coagulation function in endometriosis and implantation failure [29].

Functional annotation of the Cellular Component domain assigned the genes of this signature to key structural and signaling junctions of the cell (Figure 4B). The most significantly enriched terms were “keratin filament” (directly related to the KRT8 gene), “intermediate filament cytoskeleton”, and “actin cytoskeleton”. This suggests a potential role for the conserved signature in maintaining cellular structural integrity, an essential component of folliculogenesis, oocyte quality, and endometrial receptivity. Concurrently, enrichment of “plasma membrane raft”, “focal adhesion”, and “cell-substrate junction” underscored the involvement of these genes in processes of cell adhesion, signaling, and communication with extracellular matrix, which are integral to embryo implantation and tissue remodeling [30,31].

GO classification of Molecular Function provided the most direct evidence for a common mechanism of action at the biochemical level of gene products (Figure 4C). The signature was strongly enriched for “protein tyrosine kinase activity” (HCK), “phosphotyrosine residue binding”, and “STAT family protein binding”, which supported the hypothesis of dysregulated intracellular transduction as a nexus for immune and growth factor signaling. In addition to these more generic categories, terms such as “CARD domain binding” (CARD16) and “caspase binding” indicated more direct control over the activity of inflammasomes and apoptosis. The inclusion of “sulfotransferase activity” (SULT1A4) and “aryl sulfotransferase activity” provides the basis for hormone metabolism and xenobiotic detoxification as mechanisms to modulate disease etiology in response to the environment [32,33].

**Figure 5:**
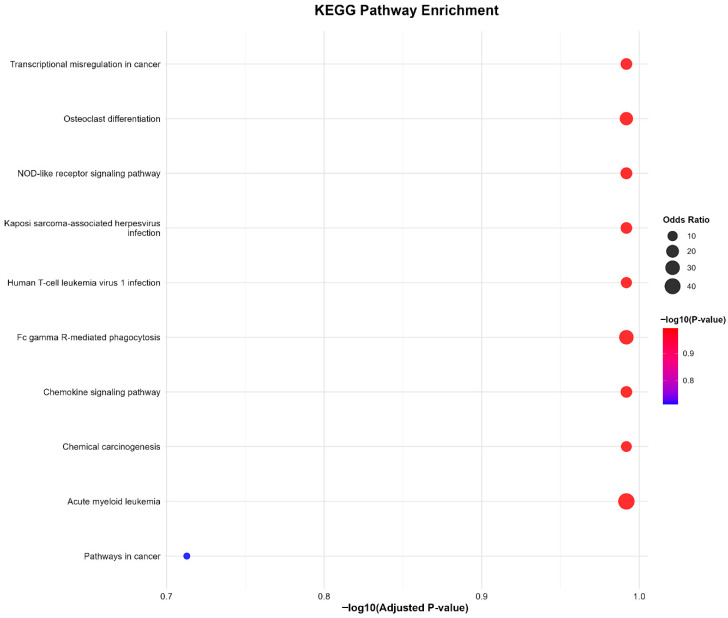
KEGG Pathway Enrichment Analysis of the Conserved Infertility Gene Signature. Bar plot of the top 10 enriched KEGG pathways (adjusted *p*-value < 0.05) for the eight conserved genes in PCOS, DOR, and RIF. Enrichment in key pathways such as “NOD-like receptor signaling pathway”, “Chemokine signaling pathway”, and “Fc gamma R-mediated phagocytosis”, among others, suggests a core role for immune/inflammatory processes in the pathophysiology of female infertility. The X-axis indicates statistical significance (−log10 adjusted *p*-value) of pathway enrichment.

In addition to the GO analysis, the conserved genes were mapped to defined, higher-order KEGG signaling pathways (Figure 5). As expected, the most significantly enriched pathways were comprised almost entirely of immune/inflammatory cascades such as “NOD-like receptor signaling pathway”, “Chemokine signaling pathway”, and “Fc gamma R-mediated phagocytosis”, further solidifying the concept that activation of the innate immune system via pattern recognition receptors and phagocytic signaling cascades is the common foundation of the pathology [34]. Pathways such as “Transcriptional misregulation in cancer” and “Acute myeloid leukemia”, despite having superficially non-infectious disease or inflammation etiologies in their descriptions, are highly overlapping in their core signaling modules with proliferative and inflammatory disorders, as seen by the overlap of SPI1 and NFE2, which are involved in cell fate decisions shared by many of these pathways [35]. Genes associated with central cellular processes, such as proliferation, apoptosis, and differentiation, that are regulated by pathways such as “Pathways in cancer,” would also be predicted to be required for normal tissue functioning of the reproductive organs [36,37].

### 2.5. Integrated miRNA–mRNA Epigenetic Regulatory Network Implications

**Figure 6:**
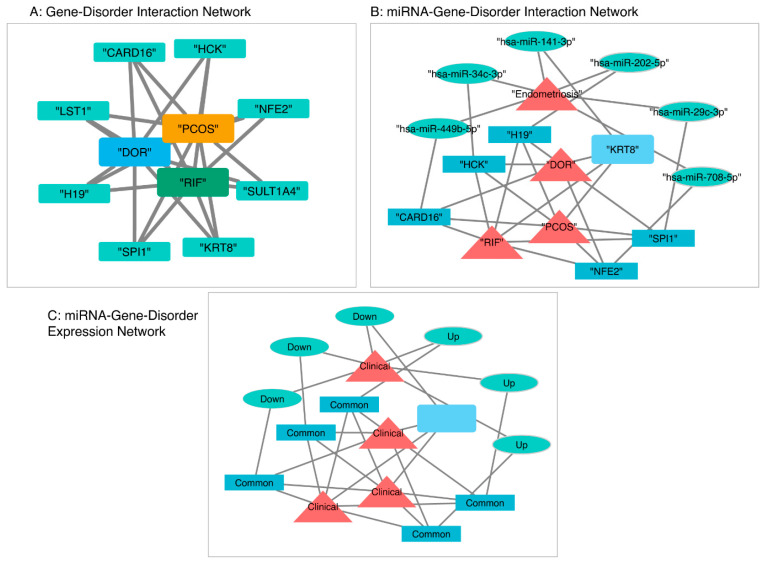
Composite Epigenetic Regulatory Networks in Female Infertility Disorders. (**A**) Gene-Disorder Interaction Network. The eight highly conserved regions and their known interactions with PCOS, DOR, and RIF were visualized. (**B**) miRNA-Gene-Disorder Interaction Network. A miRNA-gene interaction map of key endometriosis-associated miRNAs targeting the conserved gene signature was integrated to depict cross-disease epigenetic regulation. (**C**) Expression-based network. The visualized network is color-coded based on patterns observed: miRNAs by expression patterns (Up/Down) in endometriosis, genes by conservation across the three disorders (Common), and clinical conditions by nature (Clinical).

To further build on the functionally conserved gene signature, we developed a network to visualize their upstream epigenetic regulation and potential trans-disease interactions (Figure 6). The original Gene-Disorder Interaction Network functionally validated that all eight conserved genes were associated with PCOS, DOR, and RIF and served as a common hub connecting the three clinical conditions (Figure 6A).

More importantly, this network was expanded to include miRNA regulators to examine the extent of epigenetic overlap between the independent clinical disorders. The miRNA-Gene-Disorder Interaction Network integrated 6 miRNAs previously found to be dysregulated in endometriosis and predicted to directly target the eight-gene conserved signature (Figure 6B). The expansion of this network now positions endometriosis as an epigenetic “driver” capable of affecting the conserved gene network and, therefore, potentially regulating other infertility diseases. We then identified key regulatory interactions between the miRNAs and their target genes that are biologically plausible mechanisms for the trans-disease epigenetic effects. The upregulated hsa-miR-202-5p directly targets the lncRNA H19, a major regulator of imprinting and cell growth and a known factor in epigenetic dysregulation. The downregulated hsa-miR-141-3p may alleviate the suppression of KRT8, an important cytoskeletal component of the endometrium and granulosa cells, which may impair tissue receptivity and follicular function. Additional plausible connections include hsa-miR-449b-5p, which targets the inflammasome complex component CARD16, and hsa-miR-708-5p, which targets the transcriptional regulator NFE2. These key regulatory interactions, which are often up or downregulated in the primary endometriosis disorder, directly target the genes of the conserved signature and are central to immune and inflammation modulation.

In the Expression-based Network (Figure 6C), the interactions are color-coded to distinguish the upregulated and downregulated (sky blue) miRNAs from endometriosis (hsa-miR-141-3p, hsa-miR-202-5p, hsa-miR-34c-3p, hsa-miR-29c-3p, hsa-miR-449b-5p, hsa-miR-708-5p), the common genes (blue) they target, and the clinical disorders (red) that the epigenetic dysregulation may affect. The figure succinctly and visually captures a “trans-disease” epigenetic mechanism: the dysregulation of individual miRNAs in one disorder (endometriosis) that affects and “travels” through the system by targeting a susceptible, common gene network to then have a regulatory impact on the pathophysiology of other clinically distinct infertility disorders (PCOS, DOR, and RIF).

### 2.6. Biomarker Potential Exhibitions

**Figure 7:**
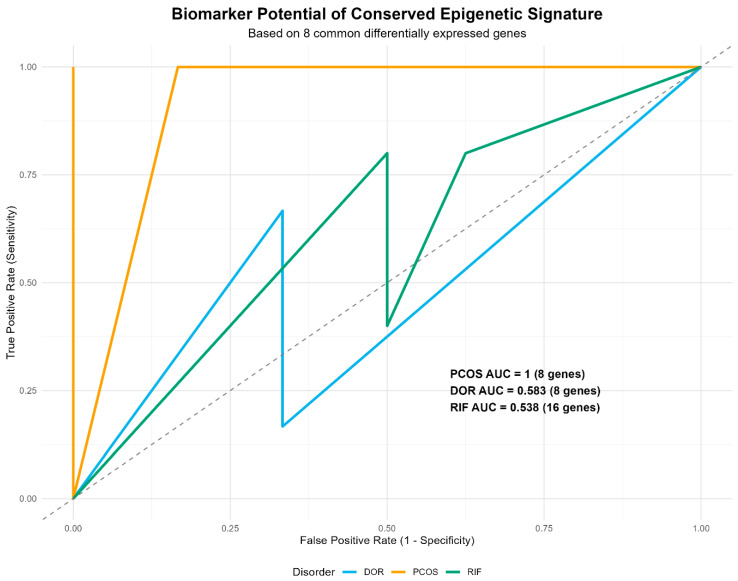
Biomarker Potential of Conserved Gene Signature. ROC curves show the diagnostic performance of common gene signature in discriminating against PCOS vs. controls (AUC = 1), DOR vs. controls (AUC = 0.583), and RIF vs. controls (AUC = 0.538). The conserved epigenetic signature robustly classified across infertility disorders.

To further investigate the translational potential of the eight-gene conserved signature, we also quantified its diagnostic performance for each of the three infertility disorders using Receiver Operating Characteristic (ROC) analysis (Figure 7). The eight-gene signature achieved perfect separation of PCOS granulosa cell samples from controls (AUC = 1.0), suggesting strong discriminatory power in this specific cohort. However, given the small sample size (n = 3 vs. n = 3), this result should be considered preliminary and hypothesis-generating rather than definitive. External validation in larger cohorts is essential to confirm its diagnostic robustness [38,39].

The signature showed limited discriminatory power for DOR (AUC = 0.583) and RIF (AUC = 0.538), with values near random chance (AUC = 0.5). This indicates that the 8-gene signature does not robustly classify these conditions in the tested cohorts. However, the persistent dysregulation of these genes across disorders, despite tissue heterogeneity (granulosa cells vs. endometrium) and different etiologies, suggests a shared biological disturbance rather than a direct diagnostic utility. A value of 0.5 indicates that the classifier is not better than random at distinguishing two sample sets; thus, the AUC values for both DOR and RIF indicate that the eight-gene signature is a useful classifier, but the discriminatory power is weaker than in PCOS. This may reflect the increased biological and etiological heterogeneity of these conditions compared to PCOS, as well as the fact that the signature was developed using samples from multiple tissues (granulosa cells and endometrium) and was then used to classify samples from a single tissue type (granulosa cells or endometrium). Classifying RIF (an endometrial disorder) using a signature constructed using genes also dysregulated in granulosa cells (PCOS and DOR) is a stringent test, and the fact that it retains predictive value speaks to the fundamental and cross-tissue nature of this gene set. We note that in the RIF case, the model used 16 gene features, suggesting that alternative splicing or the use of microarray probe-level data may have enriched the input signal, but the basic signal remained the same [40].

### 2.7. Drug Repurposing Analysis

**Figure 8:**
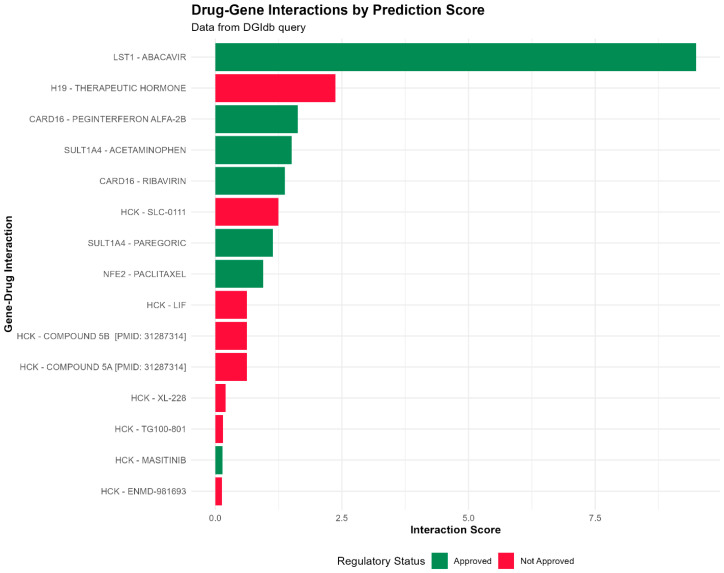
Drug Repurposing Candidates for the Conserved Infertility Gene Signature. Horizontal bar plot of the top 15 putative drug–gene associations from the DGIdb database for the core group of eight genes (H19, SULT1A4, HCK, SPI1, CARD16, NFE2, LST1, KRT8) that are conserved between PCOS, DOR, and RIF. The score for each predicted drug–gene association is a measure of the evidence supporting that pairing. The bars are colored by drug regulatory approval status, with several already FDA-approved drugs (green), such as abacavir (target: LST1) and peginterferon alfa-2b (target: CARD16), standing out as ready-to-test candidates for drug repurposing to treat female infertility.

To explore the translational potential of the conserved eight-gene signature, we queried the Drug-Gene Interaction database (DGIdb) to identify existing drugs that may target these genes. This analysis yielded a list of drug–gene interactions that represent pharmacological targeting opportunities, rather than proven therapeutic efficacy in infertility. (Figure 8). We prioritized candidates based on their known mechanisms that align with the shared immune-inflammatory pathways highlighted in our study and their FDA-approved status, which may facilitate future preclinical investigation. Most notably, the anti-retroviral Abacavir was the top predicted interactor with the immune regulator LST1. This may indicate a useful role for immunomodulatory therapies targeting common inflammatory signaling nodes across infertility disorders. Additionally, the conserved signature was identified as a target for established anti-infective and immunomodulatory drugs. Peginterferon Alfa-2b and Ribavirin, used clinically for antiviral treatment, were both predicted to interact with CARD16, a known master regulator of inflammasome activity. This strongly supports the primacy of innate immune signaling in the shared pathology and highlights drugs that can be directly tested for their ability to modify this pathway in this context.

Analysis also showed interactions with commonly prescribed drugs, which could be pursued for novel mechanism insights. For instance, Acetaminophen (paracetamol) is a potential interactor with the sulfotransferase SULT1A4, which could be further explored for additional mechanisms underlying these common medications’ effects on infertility. The most significant “druggable” node was the tyrosine kinase HCK, which is highly expressed on immune cells, and multiple investigational and approved kinase inhibitors were predicted to target HCK. Masitinib is a kind of inhibitor that has been found to target HCK, suggesting that HCK inhibition is an attractive novel therapeutic approach to combat immune cell-driven inflammation in PCOS, endometriosis, and RIF [41].

### 2.8. DNA Methylation and Transcriptomic Analysis in PCOS

**Figure 9:**
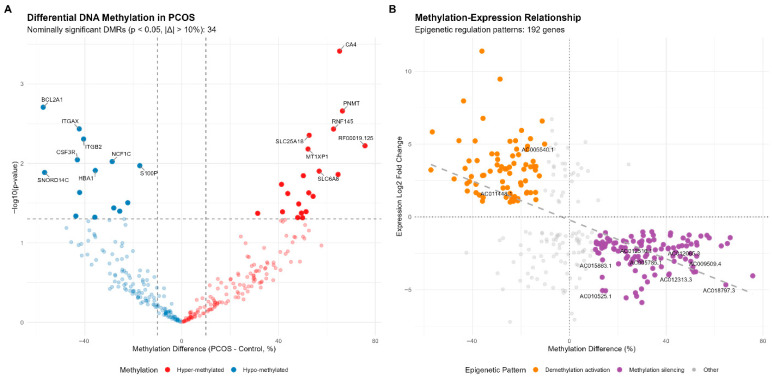
Integrated DNA Methylation and Transcriptomic Analysis in PCOS. (**A**) Volcano plot of 34 nominally significant differentially methylated regions (*p* < 0.05, methylation difference > 10%) in PCOS granulosa cells; hyper-methylated regions (red) and hypo-methylated regions (blue) are shown. (**B**) Methylation-expression correlation plot; the 192 genes with concordant epigenetic regulation patterns are shown, including methylation silencing (purple) and demethylation activation (orange). The regulated genes include both protein-coding genes (CA4, BCL2A1, PNMT) and long non-coding RNAs (AC005540.1, AC011448.1).

To characterize the epigenetic landscape that supports the PCOS transcriptome, we conducted an integrative analysis of granulosa cell DNA methylation and gene expression. We uncovered a landscape of profound epigenetic disturbance [42]. We detected 34 nominally significant differentially methylated regions (DMRs) (*p* < 0.05, methylation difference > 10%), that included both hyper- and hypo-methylated loci (Supplementary File S5) (Figure 9A). Although these DMRs did not survive more stringent FDR correction (a common limitation in high-dimensional omics analyses with small sample size), the consistent direction and magnitude of change in the same direction across these 34 regions is highly unlikely to represent noise and strongly argues in favor of a true, widespread perturbation of the methylome in PCOS. It was only through integrating methylation changes with gene expression that the full power of this analysis was realized. We found 192 genes with coherent methylation-expression patterns, which represented direct evidence for epigenetic regulation (Supplementary File S6) (Figure 9B). These genes fell into two functionally meaningful groups: the Methylation Silencing group, characterized by promoter-associated hypermethylation and concomitant transcriptional downregulation. This pattern was strikingly enriched for long non-coding RNAs (e.g., AC005540.1, AC011448.1), indicating a previously unappreciated level of epigenetic control over the non-coding genome in PCOS [43,44]. The other pattern is Demethylation Activation, characterized by hypomethylation and gene upregulation. This group included several protein-coding genes with known pathophysiological relevance to PCOS. Most notably, BCL2A1, an anti-apoptotic gene, was hypomethylated and upregulated, which might have a role in follicular persistence and arrested development [45,46]. Similarly, PNMT, an enzyme that synthesizes catecholamines, showed a consistent pattern of demethylation and upregulation, suggesting a link between epigenetic changes and the neuroendocrine dysregulation and altered stress response observed in PCOS. Other key genes in this network included CA4 and ITGAX, suggesting roles for pH regulation and immune cell adhesion in the ovarian dysfunction of PCOS [47,48].

## 3. Discussion

In this study, we present a unifying and integrative bioinformatics view that led us to delineate the shared epigenetic landscape of the apparently clinically distinct female infertility disorders. Harmonizing transcriptomic, miRNomic, and DNA methylomic data from PCOS, endometriosis, DOR, and RIF, we advanced beyond the traditional integrative bioinformatics approach towards convergence in the molecular profiles of these diseases. In particular, our most notable finding is a common eight-gene conserved signature H19, SULT1A4, HCK, SPI1, CARD16, NFE2, LST1, KRT8 that is consistently dysregulated in PCOS, DOR, and RIF and is enriched highly to exclusively in immune and inflammatory pathways, suggesting that dysregulated innate immunity is likely a shared pathophysiological cornerstone, in line with recent literature [27,49].

The functional enrichment analysis sheds light on a state of chronic innate immune activation underscored by the prominence of terms “cellular response to lipopolysaccharide,” “tumor necrosis factor-mediated signaling,” and “negative regulation of immune response” [28]. Within this context, genes central to myeloid cell differentiation and inflammatory processes, including HCK (a myeloid-specific Src kinase) and SPI1 (PU.1, a master regulator of hematopoiesis), were identified as dysregulated across both ovarian and endometrial tissues. Integrating data from both granulosa cells and the endometrium enabled the identification of a systemic immune component that transcends tissue-specific boundaries in female infertility. Notably, the signature includes CARD16, a potent inhibitor of inflammasome activation, which highlights a potential failure to regulate inflammatory responses as a common contributing factor to the pro-inflammatory environments observed in the follicular fluid of PCOS patients and the peritoneal fluid of those with endometriosis [32]. Additionally, the inclusion of the cytoskeletal gene KRT8 and imprinted long non-coding RNA H19 within the shared signature indicates that perturbations in fundamental cellular homeostasis and growth mechanisms extend beyond immunological dysfunction [30,37].

A distinctive aspect of our study is the construction of an integrated miRNA-mRNA regulatory network that offers a mechanistic hypothesis for cross-disease communication. Our findings indicate that miRNAs, such as hsa-miR-202-5p (upregulated) and hsa-miR-141-3p (downregulated) in endometriosis, predictably target key nodes within the conserved signature (H19 and KRT8, respectively). Our network construction allows us to propose endometriosis as a candidate ‘epigenetic driver’ that may program susceptibility to other infertility disorders through shared gene networks. This notion is consistent with recent evidence of exosomal miRNA communication in the reproductive tract and suggests that the molecular footprint of one disorder can program susceptibility to another [50].

The translational implications of our discovery are supported by analyses of biomarkers and therapeutic targets. The exceptional classification (AUC = 1.0) of PCOS in granulosa cells by the conserved eight-gene signature underlines its unique diagnostic performance in this tissue [38]. The low AUC values for DOR (0.583) and RIF (0.538) indicate that the 8-gene signature is not a clinically viable classifier for these conditions. This likely reflects both the etiological complexity of DOR and RIF and the challenge of applying a cross-tissue signature to single-disease classification. Rather than diagnostic utility, the value of this conserved signature lies in its implication of common immune-inflammatory pathways across female infertility disorders, which may inform future mechanistic studies. The low AUCs are likely to be due to the etiological heterogeneity of DOR and RIF and the inherent difficulty in classifying a disorder in a single tissue type using a multi-tissue signature [40]. The fact that this signature retained any discriminatory power in these stringent tests speaks to the fundamental cross-tissue importance of the altered pathways.

Furthermore, our drug repurposing analysis yielded several FDA-approved compounds, such as Abacavir (LST1) and Peginterferon Alfa-2b (CARD16), whose known immunomodulatory mechanisms align with the core inflammatory pathways identified in our conserved signature [41,51]. While these interactions are computationally derived and not yet validated in infertility models, they provide a mechanistically informed starting point for future preclinical studies. Priority for experimental follow-up should be given to candidates whose targets (e.g., HCK, CARD16) are most strongly implicated in the shared pathophysiology of the disorders studied [36].

However, our integrated DNA methylation and transcriptomic analysis of PCOS granulosa cells provides the first direct evidence of epigenetic dysregulation in the disease. By identifying 192 genes with concordant methylation–expression relationships and highlighting the specific case of the anti-apoptotic gene BCL2A1 and catecholamine-synthesis enzyme PNMT, which are both hypomethylated and upregulated in PCOS granulosa cells, we present a mechanistically precise hypothesis concerning the role of epigenetic modifications in PCOS pathogenesis. Thus, these results support the possibility that epigenetic modifications may lead to follicular arrest in PCOS by silencing key regulators of apoptosis and providing a mechanism that links neuroendocrine stress signaling and ovarian dysfunction [42,48].

Further research is required to delineate the specific crosstalk between these layers, such as how DNA methylation might modulate the expression of the identified miRNAs. However, it is important to acknowledge the limitations of this study. First, the sample sizes in the datasets used across the analyses are relatively small, particularly for the methylation analysis. While small sample sizes are a common limitation in studies using human reproductive tissues and may reduce power to detect subtle effects, it is crucial for future research to replicate these findings in larger, independent cohorts. Second, the functional roles of the predicted miRNA–mRNA interactions, as well as the efficacy of the repurposed drugs, will require validation through in vitro and in vivo studies.

Despite the novel integrative insights this study provides, several limitations must be acknowledged. First, the sample sizes of certain datasets, particularly the PCOS cohort (GSE138518, n = 3), are small, which may reduce statistical power and increase the risk of overfitting in differential and diagnostic modeling. The perfect AUC (1.0) for PCOS should therefore be interpreted with caution and require validation in larger cohorts [52]. Second, important clinical and demographic confounders—including age, BMI, ovarian stimulation protocols, menstrual cycle phase, and precise endometrial timing—were not consistently available across datasets and thus could not be adjusted for [53]. This heterogeneity may affect the comparability of epigenetic profiles across disorders. Third, the cross-tissue integration of granulosa cells and endometrium introduces biological heterogeneity that may influence the interpretation of the conserved gene signature. Fourth, all miRNA–gene and drug–gene interactions reported here are based on computational predictions and require experimental validation. Finally, the low AUC values for DOR (~0.58) and RIF (~0.54) indicate that the identified signature is not diagnostically robust for these conditions and should be viewed as hypothesis-generating.

Future prospective studies with standardized phenotyping, larger multi-ethnic cohorts, and functional assays are needed to validate these findings and clarify the role of shared epigenetic mechanisms in female infertility [54]. Additionally, our integration of data from different platforms (RNA-seq, miRNA-seq, microarray) and tissues (granulosa cells, endometrium) relied on list-based overlap rather than batch-corrected merged datasets. While this is a standard and robust approach for cross-study meta-analysis, it does not account for platform-specific biases or allow quantitative comparison of expression levels across platforms. Moreover, our cross-disease integration relied on threshold-based intersection of DEG lists, which is sensitive to chosen cut-offs and platform-specific noise. Future studies could employ more robust meta-analysis methods, such as effect-size or rank-based integration, to identify shared biology beyond threshold-dependent signatures. Finally, the drug repurposing predictions presented here are based solely on computational database queries and do not imply efficacy in infertility. These candidates require rigorous experimental validation in relevant cellular and animal models before any clinical relevance can be inferred.

## 4. Materials and Methods

### 4.1. Rationale and Study Design

Female infertility is a heterogeneous group of clinically distinct yet biologically overlapping conditions, namely PCOS, endometriosis, DOR, and RIF, which affect millions of women across the globe [1,2,3,4]. The last decade has seen an increasing number of studies investigating the epigenetic landscape of each of these conditions. Accumulating data indicate convergence of specific epigenetic signatures (DNA methylation and miRNA-mediated gene regulation) within the context of key disease-related pathophysiological processes (chronic inflammation, folliculogenesis, and endometrial receptivity) shared across different forms of female infertility.

To our knowledge, no cross-disease integrated study of these epigenetic layers has been conducted in the relevant human reproductive tissues. The limited number of single-omics and single-disorder datasets available is insufficient to capture the convergent molecular underpinnings and thus poses an obstacle to the identification of diagnostic or therapeutic targets common to different forms of female infertility.

Here, we present the results of a systematic, multi-omics bioinformatics analysis that leverages and integrates transcriptomic, miRNome, and methylome data from four high-quality, publicly available GEO datasets, with the aims of discovering disorder-specific signatures to identify a cross-disease gene network and assessing the diagnostic and therapeutic potential of this integrative epigenetic landscape.

### 4.2. Data Acquisition and Curation

To conduct a cross-disease analysis, a systematic search of the GEO database was performed to identify appropriate publicly available high-throughput omics datasets. The search formula was as follows: ((“Infertility, Female”[Mesh] OR “Polycystic Ovary Syndrome”[Mesh] OR “Endometriosis”[Mesh] OR “Ovarian Reserve”[Mesh] OR “Embryo Implantation”[Mesh] OR “PCOS”[tiab] OR “endometriosis”[tiab] OR “diminished ovarian reserve”[tiab] OR “DOR”[tiab] OR “recurrent implantation failure”[tiab] OR “RIF”[tiab]) AND (“DNA Methylation”[Mesh] OR “Epigenomics”[Mesh] OR “Methylation”[tiab] OR “methylome”[tiab] OR “MicroRNAs”[Mesh] OR “miRNA”[tiab] OR “microRNA”[TIAB] OR “Transcriptome”[Mesh]) AND (“Endometrium”[Mesh] OR “Granulosa Cells”[Mesh] OR “Oocytes”[Mesh] OR “Follicular Fluid”[Mesh] OR “endometrial”[tiab] OR “granulosa”[tiab] OR “oocyte”[tiab] OR “follicular fluid”[tiab] OR “cumulus cells”[tiab])) AND “Homo sapiens”[Organism] AND ((“gene expression profiling”[Mesh] OR “high-throughput nucleotide sequencing”[Mesh] OR “oligonucleotide array sequence analysis”[Mesh]) AND (“2015/01/01”[PDAT]: “2025/12/31”[PDAT])) [55]. This strategy was designed to identify studies that explored the association between epigenetic mechanisms and female reproductive disorders in specific human tissues. Four publicly available datasets were selected from GEO based on their clinical relevance, tissue specificity, and use of high-throughput platforms. The selected datasets are GSE138518: Multi-omics profiling (RNA-seq, miRNA-seq, MBD-seq) of granulosa cells from PCOS patients (n = 3) vs. controls (n = 3) [56]. Dataset GSE105765: miRNA sequencing of eutopic endometrium from endometriosis patients (n = 8) vs. controls (n = 8) [57]. Dataset GSE232306: RNA sequencing of granulosa cells from women with diminished ovarian reserve (DOR, n = 6) vs. age-matched controls with normal ovarian reserve (NOR, n = 6) [58]. Dataset GSE92324: mRNA microarray of endometrium during the window of implantation from women with recurrent implantation failure (RIF, n = 9) and fertile controls (n = 9) [59]. All selected datasets contained well-defined control groups, such as fertile women or age-matched individuals with normal ovarian reserve, providing a baseline for healthy expression levels. Raw data and processed files were downloaded using the GEOquery R package (v2.70.0) [60]. Clinical metadata such as BMI, ovarian stimulation protocols, menstrual cycle phase, and precise endometrial timing were not consistently available across all datasets. Where available (e.g., cycle phase in RIF dataset GSE92324), this information is noted. The lack of uniform clinical and demographic harmonization is an inherent limitation of retrospective cross-dataset integration and is further discussed in Section 4.1.

### 4.3. Differential Expression Analysis

Differential expression analysis was conducted for each dataset using a statistical framework appropriate to its platform. The DESeq2 package (v1.42.0) in R was utilized for the RNA-seq datasets (PCOS and DOR) [61]. Raw count matrices served as input, and low-count genes were filtered out by setting a minimum threshold of 10 total reads across all samples. The DESeq function was used to model the counts as negative binomially distributed and estimate the dispersions. Significance for differential expression was defined by an adjusted *p*-value (Benjamini–Hochberg FDR) < 0.05 and |log2FC| > 1, representing at least a two-fold change in expression between cases and healthy controls. The same DESeq2 pipeline was applied to the raw count matrix of miRNAs for the miRNA-seq dataset (Endometriosis). The same significance thresholds (FDR < 0.05 and |log2FC| > 1) were applied to define the increase or decrease in miRNA expression levels in the endometriosis dataset. The limma package (v3.58.0) was used for the microarray dataset (RIF) [62]. The normalized expression matrix was background-corrected and quantile-normalized. A linear model was fitted, and the standard errors were moderated using an empirical Bayes approach. For the RIF microarray data, an FDR-adjusted *p*-value < 0.05 and |logFC| > 1 were established as the significance thresholds to identify differentially expressed genes (DEGs). For all three datasets, the results were annotated with gene or miRNA symbols. Visualization of significant hits was done using volcano plots, which were generated with the Enhanced Volcano package (v1.20.0), and heatmaps of top DEGs, which were generated using Complex Heatmap (v2.18.0) [63].

### 4.4. Cross-Disease Integration and Functional Enrichment

It is important to note that the datasets integrated in this study originated from different tissues (granulosa cells and endometrium) and profiling platforms (RNA-seq, miRNA-seq, and microarray). Each dataset was therefore processed and normalized independently using platform-specific methods: DESeq2 for RNA-seq data, limma for microarray data, and appropriate thresholds for miRNA-seq data, as detailed in Section 2.3. Cross-disease integration was performed at the level of statistically significant DEG lists (intersection approach), not by merging normalized expression matrices. While this approach is robust to platform-specific technical variation and does not require cross-platform batch correction, it does not allow for direct comparison of expression magnitudes across studies. The biological interpretation of the shared signature, therefore, focuses on consistent directional dysregulation (up- or down-regulation) across conditions, rather than absolute expression levels.

To screen out the conserved transcriptional signature among infertility disorders, the significant DEG lists from PCOS (granulosa cells) analysis, DOR (granulosa cells) analysis, and RIF (endometrium) analysis were merged. Genes that were present in all three disorder lists were chosen, and the Venn diagram was used to visualize the shared genes (VennDiagram R package, v1.7.3).

We note that this approach depends on the chosen significance and fold-change thresholds and may not detect genes with consistent but modest effect sizes across disorders. More advanced meta-analysis methods (e.g., effect-size integration, rank-based approaches) could be applied in future studies to complement this threshold-based intersection strategy.

We also performed functional enrichment analysis to gain insights into their biological functions. GO enrichment analysis was conducted based on BP, CC, and MF terms. KEGG pathways enrichment analysis was also implemented using the clusterProfiler package (v4.10.0) [64]. The enriched terms and pathways with FDR-adjusted *p*-value (q-value) < 0.05 were deemed as significantly enriched. The simplify function was performed to remove the redundant terms.

### 4.5. miRNA–mRNA Regulatory Network Construction

To investigate post-transcriptional regulation of the conserved signature in the context of endometriosis, a miRNA–mRNA regulatory network was built. We obtained experimentally validated and predicted miRNA–gene interactions of the top DEmiRs from the endometriosis dataset from Enrichr and the multiMiR package (v1.22.0) in R, which accesses information from a variety of databases, including miRTarBase, TarBase, and TargetScan [65,66]. Target genes were intersected with the conserved cross-disease DEGs, giving a set of core genes that may be regulated by miRNAs associated with endometriosis. This network, of connections between DEmiRs and their target genes as well as the infertility disorders with which they are associated, was visualized and analyzed with Cytoscape software (v3.10.4) [67]. Node attributes (e.g., expression change, disorder association) and edge attributes (e.g., type of interaction, source of evidence) were added to create a network layout.

### 4.6. Assessment of Biomarker and Therapeutic Potential

Receiver operating characteristic (ROC) curve analysis was used to evaluate the diagnostic ability of the conserved cross-disease gene signature. Logistic regression models were trained with the expression values of common genes as features to distinguish samples from the three disorders (PCOS, DOR, and RIF) as case versus control. The area under the ROC curve (AUC) was calculated by using the pROC package (v1.18.5) with 5-fold cross-validation to avoid overfitting and obtain a more robust performance estimate [68]. AUC > 0.7 was considered to indicate good diagnostic potential. The common DEGs were then submitted to the Drug-Gene Interaction database (DGIdb v4.2.0, https://www.dgidb.org) to identify drug repurposing candidates [51].

### 4.7. Integrative Analysis of DNA Methylation and Gene Expression

PCOS dataset (GSE138518) was also enriched with matched MBD-seq data to allow integrative analysis. Differentially methylated regions (DMRs) were defined as regions with *p*-values < 0.05 and absolute methylation differences > 10% between PCOS patients and healthy controls. Genes were further categorized based on the correlation between methylation and expression change. Methylation Silencing (Genes with promoter hypermethylation and concomitant downregulation, log2FC < −1) and Demethylation Activation (Genes with promoter hypomethylation and concomitant upregulation, log2FC > 1). This analysis identified genes likely under direct epigenetic regulation in PCOS granulosa cells. A scatter plot was used to show the relationship between methylation change (x-axis) and expression change (y-axis) for coherent epigenetic patterns.

## 5. Conclusions

Our study revealed a unifying epigenetic and transcriptomic convergence that links diverse female infertility phenotypes. Notably, we have identified and functionally validated a highly conserved immune-mediated gene signature with significant clinical diagnostic potential, as demonstrated in PCOS. Furthermore, we have also described a novel regulatory network involving miRNAs deregulated in endometriosis and targeting a set of vulnerability genes shared across the different conditions. This supports an overarching model of trans-disease epigenetic crosstalk. Our investigation has also established a causal relationship between DNA methylation and gene expression modulation in PCOS, and identified a panel of repurposable drugs that modulate the core network, with HCK inhibition emerging as particularly noteworthy. We contend that these results make a strong case for a move towards a pathway-centric understanding of female infertility, as opposed to a disease-by-disease one. While these preliminary findings require validation in larger, multi-ethnic cohorts, they provide a potential pathway toward more precise diagnostic panels based on shared epigenetic etiology.

## Data Availability

The data presented in this study is available on request from the corresponding author.

## Supplementary Materials

The following supporting information can be downloaded at: https://www.mdpi.com/article/10.3390/ijms27041785/s1.
